# Changes in university student health behaviors since the beginning of the COVID-19 pandemic: Marie-Pierre Tavolacci

**DOI:** 10.1093/eurpub/ckac131.318

**Published:** 2022-10-25

**Authors:** A Patin, J Ladner, MP Tavolacci

**Affiliations:** 1 CIC 1404, CHU Rouen, Rouen, France; 2 DEPS, CHU Rouen, Rouen, France; 3 INSERM 1073, Université de Rouen, Rouen, France

## Abstract

**Background:**

The COVID-19 pandemic lead many upheavals in the life habit whith lockdowns and curfews since March 2020 and possibly in the health behaviours of university students could be impacted. The objective of the study was to assess the evolution of the health behaviors of university students between before the COVID-19 period and May 2021.

**Methods:**

Two retrospectives online studies of a university students in France were in May 2020 and May 2021 were conducted. Socio demographics and academics environment data, tobacco smoking, binge drinking, cannabis use, and vigorous physical activity were collected in a declarative way.

**Results:**

In 2020, 3483 (72.5% of women, mean age 20.9 (SD = 2.46)) and in 2021, 3504 (74.4 of women, mean age 20.73 (SD = 2.32)) university students were included. After logistic regression, in 2020 compared to the pre-COVID19 period, the regular vigorous physical activity didn't change significantly while there appears to be a study period effect with a decrease of the regular binge drinking (AOR=0.24 IC95% [0.20,0.29]). In 2021 compared to the pre-COVID19 period, the regular vigorous physical activity and the regular binge drinking decreased respectively, (AOR=0.53; IC95% [0.48,0.59]) and (AOR=0.60 IC95% [0.52,0.70]). Regular tobacco smoking and cannabis use didn't change significantly in 2020 and 2021 compared to the pre-COVID period.

Discussion: The decrease of binge drinking and physical activity between preCOVID-19 and 2021 could be attribute by the restricting mobility and social interactions. We did not observe an increase in risky health behaviors as smoking and cannabis use. These behaviors remain to be monitored in the future to assess the long-term effects of the pandemic COVID-19 on student health behaviors

**Key messages:**

• Health-promotion strategies directed at adopting or maintaining positive mental health should be developed for university students to better manage future lockdown periods.

• Recommendations to maintain health during the ongoing COVID-19 pandemic specifically target university student populations are needed.

